# A DNA adductome analysis revealed a reduction in the global level of C5-hydroxymethyl-2′-deoxycytidine in the non-tumoral upper urinary tract mucosa of urothelial carcinoma patients

**DOI:** 10.1186/s41021-021-00228-9

**Published:** 2021-12-02

**Authors:** Yuto Matsushita, Yuji Iwashita, Shunsuke Ohtsuka, Ippei Ohnishi, Takashi Yamashita, Hideaki Miyake, Haruhiko Sugimura

**Affiliations:** 1 Department of Tumor Pathology, Hamamatsu University School of Medicine, 1-20-1 Handayama, Higashi-ku, Hamamatsu, Shizuoka, 431-3192 Japan; 2 Department of Urology, Hamamatsu University School of Medicine, 1-20-1 Handayama, Higashi-ku, Hamamatsu, Shizuoka, 431-3192 Japan

**Keywords:** DNA adduct, DNA adductome, DNA adductomics, C5-hydroxymethyl-2′-deoxycytidine, Renal cell carcinoma, Upper urinary tract urothelial carcinoma, Oxidative DNA damage

## Abstract

**Background:**

DNA adducts, covalent modifications to DNA due to exposure to specific carcinogens, cause the mispairing of DNA bases, which ultimately results in DNA mutations. DNA methylation in the promoter region, another type of DNA base modification, alters the DNA transcription process, and has been implicated in carcinogenesis in humans due to the down-regulation of tumor suppressor genes. Difficulties are associated with demonstrating the existence of DNA adducts or chemically modified bases in the human urological system. Apart from aristolochic acid-DNA adducts, which cause urothelial carcinoma and endemic nephropathy in a particular geographical area (Balkan), limited information is currently available on DNA adduct profiles in renal cell carcinoma and upper urinary tract urothelial carcinoma, including renal pelvic cancer and ureteral cancer.

**Method:**

To elucidate the significance of DNA adducts in carcinogenesis in the urothelial system, we investigated 53 DNA adducts in the non-tumoral renal parenchyma and non-tumoral renal pelvis of patients with renal cell carcinoma, upper urinary tract urothelial carcinoma, and other diseases using liquid chromatography coupled with tandem mass spectrometry. A comparative analysis of tissue types, the status of malignancy, and clinical characteristics, including lifestyle factors, was performed.

**Results:**

C5-Methyl-2′-deoxycytidine, C5-hydroxymethyl-2′-deoxycytidine (5hmdC), C5-formyl-2′-deoxycytidine, 2′-deoxyinosine, C8-oxo-2′-deoxyadenosine, and C8-oxo-2′-deoxyguanosine (8-OHdG) were detected in the renal parenchyma and renal pelvis. 8-OHdG was more frequently detected in the renal pelvis than in the renal cortex and medulla (*p* = 0.048 and *p* = 0.038, respectively). 5hmdC levels were significantly lower in the renal pelvis of urothelial carcinoma patients (*n* = 10) than in the urothelium of patients without urothelial carcinoma (*n* = 15) (*p* = 0.010). Regarding 5hmdC levels in the renal cortex and medulla, Spearman’s rank correlation test revealed a negative correlation between age and 5hmdC levels (*r* = − 0.46, *p* = 0.018 and *r* = − 0.45, *p* = 0.042, respectively).

**Conclusions:**

The present results revealed a reduction of 5hmdC levels in the non-tumoral urinary tract mucosa of patients with upper urinary tract urothelial carcinoma. Therefore, the urothelial cell epithelia of patients with upper urinary tract cancer, even in non-cancerous areas, may be predisposed to urothelial cancer.

**Supplementary Information:**

The online version contains supplementary material available at 10.1186/s41021-021-00228-9.

## Introduction

The initiation of carcinogenesis involves the induction of irreversible genetic alterations, including mutations, transversions, transitions, and/or deletions in DNA. One of the causes of DNA mutations is DNA adducts, which are covalent modifications to DNA due to exposure to specific carcinogens. Another cause is modified DNA bases, which are enzymatically generated in the human body [1]. Some DNA base modifications in promoter regions (adducts in a broad sense), such as C5-methyl-2′-deoxycytidine (5mdC), alter the DNA transcription process, which regulates gene expression. Promoter methylation in tumor suppressor genes has been implicated in carcinogenesis and the poor prognosis of some cancers [2–5]. DNA adducts and modified DNA bases, which can be detected in analytical chemistry using tissue, urine, and blood, have been examined as biomarkers in individual assessments of the risk of developing cancer. Therefore, the analysis of DNA adducts and modified DNA bases may eventually lead to the early detection of cancer [6].

DNA adducts in human tissue have been investigated as the cause of DNA mutations in the lungs and gastrointestinal tract, which are directly exposed to potential procarcinogens and mutagens derived from environmental substances, such as tobacco smoke, polluted air, alcohol, and dietary carcinogens. Thus, the DNA lesions caused by these environmental constituents have been detected in the lungs and gastrointestinal tract [7–10]. The urinary system, which is constantly exposed to urine containing hydrophilic phase II metabolites, is another organ that is exposed to many environmental mutagens. Urinary C8-oxo-2′-deoxyguanosine (8-OHdG) has been investigated as a biomarker of oxidative DNA damage [11]. However, limited information is currently available on other DNA adducts in human urological organs, including the kidneys, ureter, and bladder [12–16]. Aristolochic acid (AA)-DNA adducts and their role in the development of urothelial carcinoma (UC), renal cell carcinoma (RCC), and nephropathy in endemic areas and Chinese herb users [17] have recently been attracting increasing attention [18–22]. The mechanisms underlying carcinogenesis induced by AA have been investigated in vitro and in vivo [23]. Smoking has been identified as the most important risk factor for UC, particularly bladder cancer [24]. DNA adducts induced by aromatic amines, such as 4-aminobiphenyl from tobacco smoke, have been detected in the non-tumor bladder tissues of patients with bladder cancer and interactions with genetic polymorphisms were reported [12, 13].

The roles of the hydroxymethylation of cytosine, detected as C5-hydroxymethyl-2′-deoxycytidine (5hmdC) or 5-hydroxymethylcytosine (5hmC), in cancer are currently being investigated. A previous study demonstrated that 5hmdC levels were lower in malignant tumor tissue, including clear cell RCC and UC, than in normal tissue [25]. 5hmC functions as a cis-regulatory element in transcription factor-binding regions. Furthermore, it may associate with histone modifications to alter the configuration of chromatin and modulate alternative splicing [26]. 5hmC has also been shown to maintain the integrity of stalled replication forks and has been linked to resistance to poly (ADP-ribose) polymerase inhibitors and cisplatin [27].

Although various types of known and unknown noncanonical deoxyribonucleosides may be involved in urinary carcinogenesis, their role remains unclear [12, 16, 25]. A more detailed understanding of DNA adducts and modified DNA bases, particularly in the human renal parenchyma and renal pelvis, will provide important insights into the etiologies and carcinogenesis of RCC and upper urinary tract urothelial carcinoma (UTUC). Therefore, our research has focused on DNA adducts and modified DNA bases in the non-tumoral tissue of urological organs.

We previously detected lipid peroxidation-induced DNA adducts in the non-tumoral tissue of gastric cancer patients [8]. This finding prompted us to investigate whether these adducts are also present in urological organs. RCC originates from the renal cortex and UTUC from the urothelium of the renal pelvis and ureter. Therefore, we herein compared the DNA adduct profiles of the renal parenchyma obtained from patients with and without RCC and those with and without UTUC.

## MATERIALS and METHODS

### Tissue collection

**Fig. 1:**
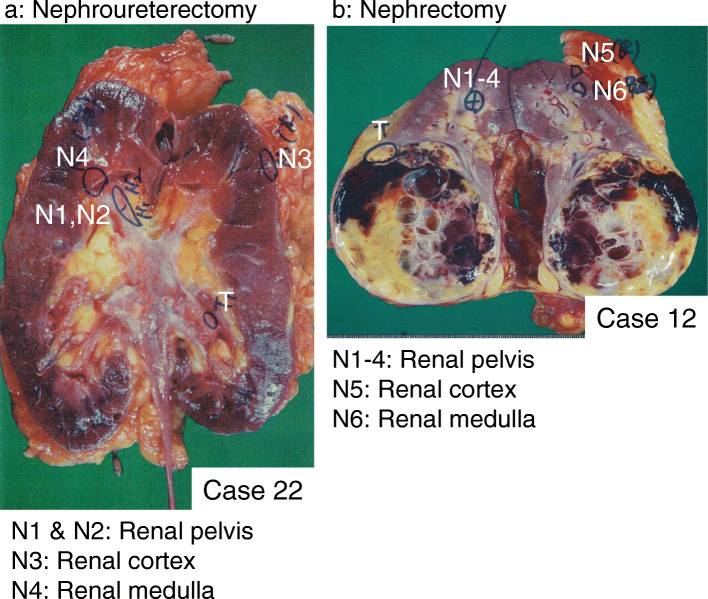
Examples of sampling sites from resected kidneys. **a** A macroscopic view of multisite sampling in a kidney from a patient with renal pelvic cancer who underwent nephroureterectomy. The normal renal pelvic mucosa (N1 and N2) was taken from regions other than the tumor (T). Samples of the renal cortex (N3) and renal medulla (N4) were also collected. **b** The sites of tissue sampling in a kidney with renal cell carcinoma (T). Renal pelvis tissues were collected and named N1 to N4. Samples of the renal cortex (N5) and renal medulla (N6) were taken from the non-tumoral renal parenchyma

Sample tissues were obtained from consecutive cases of RCC, UTUC, or other benign diseases, including oncocytoma and urinary lithiasis, that underwent nephrectomy and nephroureterectomy at Hamamatsu University Hospital. The renal cortex, renal medulla, and urothelial mucosa of the renal pelvis were collected as the non-tumoral tissue of resected kidneys. Representative cases are shown in Fig. 1. Tissues were also collected from autopsy cases of duodenal ulcers, germ cell tumors, interstitial pneumonia, pleomorphic sarcoma, and lung cancer. Tissues were obtained from 49 patients (26 renal cortex tissue samples from 14 RCC patients [the cortex-RCC group] and 12 non-RCC patients [the cortex-non-RCC group], including 8 UTUC cases, 2 non-malignant cases, one ovarian cancer case, and one sarcoma case; 21 renal medulla tissue samples from 12 RCC patients [the medulla-RCC group] and 9 non-RCC patients [the medulla-non-RCC group], including 8 UTUC cases and one non-malignant case; 25 samples of the urothelium of the renal pelvis from 10 UTUC patients [the pelvis-UTUC group] and 15 non-UTUC patients [the pelvis-non-UTUC group], including 10 RCC cases and 5 cases without urological malignancies). Clinicopathological features and tissue types are shown in Table 1. In several cases, two or three portions were collected from individual patients. For example, sample No.1 from the renal cortex and sample No.29 from the renal medulla were taken from the same patient. Details on the kidney samples collected, including clinicopathological features, are shown in Supplementary Table S1. Kidney samples obtained immediately after resection or autopsy were frozen in liquid nitrogen and stored at − 80 °C in a deep freezer until DNA extraction. The locations of sampled portions in the kidneys were photographed to ensure that tumorous tissues were not contaminated in the sampled tissues. All procedures to collect tissue samples were performed according to the principles guided by the Japanese Society of Pathology [28].

**Table 1:** Clinicopathological characteristics

Tissue	Renal cortex			Renal medulla			Renal pelvis		
Group	RCC	non-RCC	*p*	RCC	non-RCC	*p*	UTUC	non-UTUC	*p*
N	14	12		12	9		10	15	
Age									
≥ 70	6	9	0.13	6	5	1.00	5	7	1.00
< 70	8	3		6	4		5	8	
Sex									
Male	10	9	1.00	9	8	0.60	8	11	1.00
Female	4	3		3	1		2	4	
Smoking history									
Current smoker	3	1*	0.86	3	0	0.24	2	2	0.76
Ex-smoker	8	6*		3	5		4	5	
Non-smoker	3	3*		6	4		4	8	
Herb use									
Yes	0	3	0.085	0	0	NA	2	0**	0.16
No	14	9		12	9		8	14**	
eGFR (mL/min)									
> 60	9	3	0.062	8	4	0.40	4	10**	0.38
≤ 60	5	9		4	5		5	4**	
T category***									
Tis	–	–	–	–	–	–	1	–	–
T1	2	–	–	2	–	–	3	–	–
T2	0	–	–	0	–	–	2	–	–
T3	12	–	–	10	–	–	4	–	–

The basic characteristics of patients are summarized. Tumor-related information is also presented for RCC and UTUC cases. This information is described in detail in Supplementary Table S1. *P* values were calculated using Fischer’s exact test. *Information was not available for two cases. ** Information was not available for one case. ***The T category is according to the WHO Classification of Tumours, 4th Edition

### DNA extraction from kidney tissue samples and enzymatic digestion

DNA extraction was conducted on all tissue samples using a Gentra Puregene™ Tissue Kit (Qiagen, Valencia, CA) according to the manufacturer’s instructions. Using a median tissue mass of 110 mg (range, 27–296 mg), we purified a median of 93.4 μg DNA (range, 41.3–526.3 μg). Purified DNA was quantified by measuring absorbance at 260 and 280 nm with a Nano Drop ND-1000 spectrophotometer (Thermo Fisher Scientific, Tokyo, Japan). To prevent the oxidation of DNA, deferoxamine was added to the DNA solvent at a final concentration of 0.1 mM. DNA was prepared for liquid chromatography coupled with tandem mass spectrometry (LC/MS) using an 8-OHdG Assay Preparation Reagent Set (Wako, Osaka, Japan). Ten micrograms of DNA was transferred to a 1.5-mL Eppendorf tube and diluted to 52.5 μL using distilled water. After samples had been incubated at 98 °C for 2 min and placed on ice for 5 min, 6.7 μL of Acetic Acid Buffer and 3.5 μL of Nuclease P1 were added to the mixture, which was then incubated at 37 °C for 30 min. Following the addition of 7.0 μL of Tris Buffer and 0.35 μL of Alkaline Phosphatase Solution, samples were incubated at 37 °C for 30 min. The solution was centrifuged at 15000×*g* at 20 °C for 15 min using Nanosep 10 K (Pall Life Science, Michigan, USA) to filter enzymatically hydrolyzed deoxyribonucleoside monomers.

### DNA adduct identification and semi-quantification by LC/MS

A 4000 QTRAP mass spectrometer (SCIEX, Framingham, MA, USA) with an Acquity UPLC system (Waters, Milford, MA, USA) was used to analyze DNA adducts. An aliquot of a digested DNA sample (1.1 μg/7.5 μL) was injected and separated using an Acquity UPLC HSS T3 column (2.1 × 100 mm, Waters) with mobile phase A: 0.02% (v/v) acetic acid in water and mobile phase B: methanol at 0.2 mL/min with an isocratic and linear gradient elution program (3% B from 0 to 5 min; 3–15% B from 5 to 10 min; 15–80% B from 10 to 25 min; 80% B from 25 to 30 min). Other experimental conditions were set as follows: ion source temperature, 400 °C; ion spray voltage, 5500 V; declustering potential, 51 V; entrance potential, 15 V; collision energy, 18 eV; collision cell exit potential, 6 V. Selected reaction monitoring in the positive ion mode was designed to mainly detect the neural loss of 2′-deoxyribose from positively ionized 2′-deoxynucleoside adducts ([M + H]^+^ → [M + H-116]^+^). To enhance detection, we limited and scheduled the dwell time for counting ions at the column retention time of standard compounds according to each mass transition. We visually evaluated chromatograms and identified known DNA adducts and modifications, which are described in Supplementary Table S2. Distilled water and a “blank sample” in which distilled water was used instead of DNA in the enzymatic digestion procedure were used in every measurement to monitor the background. A “DNA sample”, consisting of 24.5% 2′-deoxyadenosine (dA), 24.5% 2′-deoxythymidine (dT), 20.5% 2′-deoxyguanosine (dG), and 20.5% 2′-deoxycytidine (dC) at 1.0 μg/mL, and a “standard sample” containing all the standard compounds we prepared (Supplementary Table S2), were used to identify each DNA adduct and monitor day-to-day variance.

### Data processing

Acquired wiff files were analyzed using Skyline software v20.1.0.155 (https://skyline.ms). The peak boundary of each adduct was defined as the minimum and maximum column retention times based on five measurements using the “standard sample”. Chromatograms were visually evaluated to exclude apparent peaks with a deviation of more than 0.2 min from the authentic retention time of the “standard sample” as well as narrower peaks, which were suspected to be artifacts. We set the detection limit as the peak area, which was less than the mean values of five “blank sample” measurements. We surveyed 53 types of DNA adducts and modifications and characterized the following 6 molecules: 5mdC, 5hmdC, C5-formyl-2′-deoxycytidine (5fdC), 2′-deoxyinosine (dI), C8-oxo-2′-deoxyadenosine (8-OHdA), and 8-OHdG, from kidney samples. Raw data on the areas of these 6 adducts and 4 isotopologues of deoxyribonucleosides are shown in Supplementary Table S3.

Raw area data were normalized by the quantity of the naturally-occurring isotopologues of deoxyribonucleosides detected in each sample, such as ^13^C_2_-dA, ^13^C_2_-dT, ^13^C_2_-dG, and ^13^C_2_-dC. To adjust intra-day and day-to-day differences, the areas of these four isotopologues in kidney samples were divided by the median value of the corresponding isotopologue in all measurements. The median of the ratios of these four isotopologues in a sample was defined as the “correction coefficient” of each sample. In our experiments, the “correction coefficient” ranged between 0.43 and 1.60. To normalize the area data of kidney samples, we divided each area of kidney samples by the “correction coefficient” and calculated the “normalized area” (Supplementary Table S4).

To convert “normalized area” data into a molar ratio, we used “mol/area ratio” calculated by the molecular weight of each DNA adduct and the average area of the standard sample with a defined amount. The “mol/area ratios” of 5mdC, 5hmdC, 5fdC, dI, 8-OHdA, and 8-OHdG were 3.58 × 10^− 19^, 5.10 × 10^− 19^, 6.55 × 10^− 19^, 3.95 × 10^− 19^, 1.34 × 10^− 18^, and 8.58 × 10^− 19^, respectively. The molar quantity of DNA adducts in kidney samples was acquired as a product of the “normalized area” and “mol/area ratio” and was divided by the number of molecules of intact deoxyribonucleoside calculated from the genome DNA amount and composition with GC contents 41% to acquire the molar ratio (Supplementary Table S5). Data were shown with three-digit accuracy.

### Collection of clinical data

Electronic medical records were reviewed to obtain variables including age, sex, weight, laboratory data, and pathological diagnosis. Information on smoking habits and a history of taking herbal medicine was also retrospectively collected. The estimated glomerular filtration rate (eGFR) was calculated using the Cockcroft-Gault equation: in males, eGFR = (140 - age) × weight (kg)/72 × plasma creatinine (mg/dL); in females, eGFR = 0.85 × (140 - age) × weight (kg)/72 × plasma creatinine (mg/dL).

### Statistical analysis

The number of samples in which each DNA adduct was detected was compared by Fisher’s exact test between 3 tissue types: the renal cortex, renal medulla, and renal pelvis. The Mann–Whitney U test was performed to compare the molar ratio of DNA adducts in the following pairs: the cortex-RCC group and cortex-non-RCC group, the medulla-RCC group and medulla-non-RCC group, and the pelvis-UTUC group and pelvis-non-UTUC group. The relationships between each DNA adduct and clinical characteristics, including age, sex, smoking history, herb usage, eGFR, and the status of malignant tumors were assessed. Continuous variables, including age and eGFR, were analyzed by Spearman’s rank correlation test and other dichotomous variables were examined using the Mann–Whitney U test. Patients were classified as non-smokers [smoking history (−)] and ex-smokers and current smokers as smokers [smoking history (+)]. In pelvis-UTUC group, extended analyses were performed to compare the quantity of 5hmdC between solitary UTUC and multiple UTUC, and between UTUC with history of bladder cancer and UTUC without it using the Mann–Whitney U test. All calculations were performed using SPSS (IBM Corp. Released 2019. IBM SPSS Statistics for Windows, Version 26.0. Armonk, NY: IBM Corp). The significance of differences in the present study was set at *p* < 0.05 and all reported *p* values were two-sided.

## Results

**Fig. 2:**
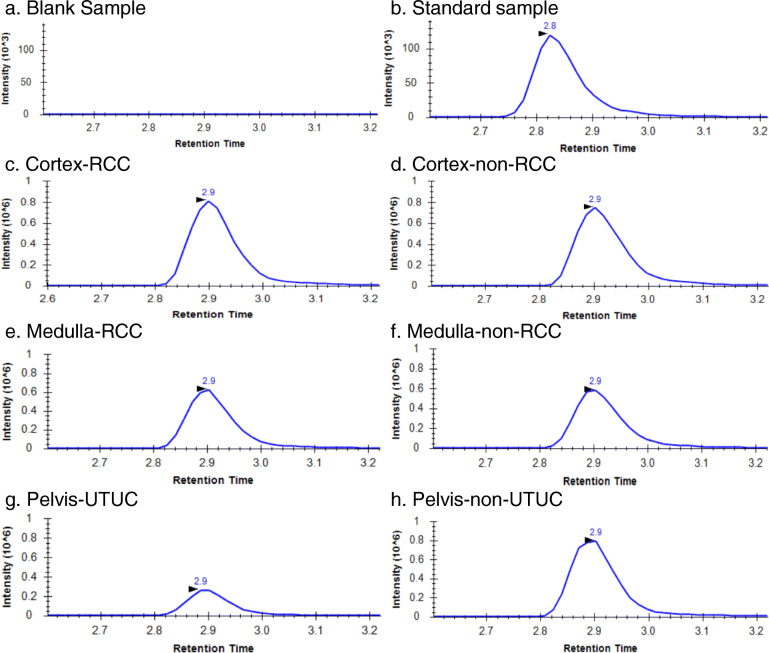
Representative chromatograms of 5hmdC. The x-axis represents the retention time. The y-axis represents the intensity of the target molecule and the arrowhead shows the “best retention time” corresponding to the highest peak in the peak boundary. A blank sample (**a**) and standard sample (**b**) that contained a chemical compound of 5hmdC were used as controls. Representative chromatograms are shown from every group (**c**: Cortex-RCC group, **d**: Cortex-non-RCC group, **e**: Medulla-RCC group, **f**: Medulla-non-RCC group, **g**: Pelvis-UTUC group, **h**: Pelvis-non-UTUC group). The best retention times of all groups were consistent

Figure 2 shows representative chromatograms of 5hmdC in the “standard sample”, “blank sample”, and kidney samples of every group. After adopting the defined threshold, the prevalence of 5mdC, 5hmdC, 5fdC, dI, 8-OHdA, and 8-OHdG is shown in Table 2. 5mdC, 5hmdC, and dI were detected in all kidney samples. As shown in Supplementary Table S2, although C5-carboxy-2′-deoxycytidine (5cadC) was investigated, its levels were below the threshold. Fisher’s exact test revealed differences in the prevalence of the 6 molecules between tissue types, with 8-OHdG being more frequently detected in the renal pelvis than in the renal cortex and medulla (*p* = 0.048 and *p* = 0.038, respectively). The *p* values of the other pairs were > 0.05 (Supplementary Table S6). The prevalence of 5mdC, 5hmdC, 5fdC, dI, 8-OHdA, and 8-OHdG was also compared among the following cohort pairs: the cortex-RCC group and cortex-non-RCC group, medulla-RCC group and medulla-non-RCC group, and pelvis-UTUC group and pelvis-non-UTUC group. However, the *p* values of these pairs were > 0.05 (Supplementary Table S6).

**Table 2:** Sample numbers and percentages of each DNA adduct by tissue types

	Renal cortex *n* = 26	Renal medulla *n* = 21	Renal pelvis *n* = 25
5mdC	26 (100%)	21 (100%)	25 (100%)
5hmdC	26 (100%)	21 (100%)	25 (100%)
5fdC	5 (19%)	1 (4.8%)	5 (20%)
dI	26 (100%)	21 (100%)	25 (100%)
8-OHdA	4 (15%)	2 (9.5%)	1 (4.0%)
8-OHdG	7 (27%)	5 (24%)	14 (56%)

**Fig. 3:**
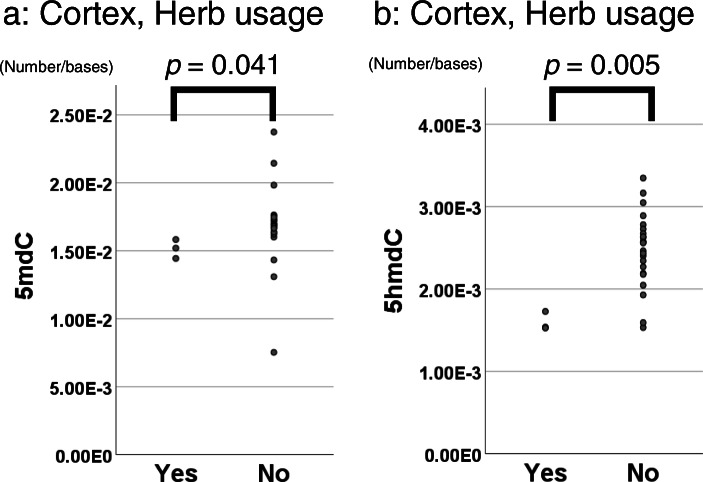
Relationships between herb usage and DNA adducts. The relationships between herb usage and 5mdC (**a**) and 5hmdC (**b**) levels in the renal cortex are shown as dot plots. The y-axis represents the molar ratio (number/base) of each DNA adduct

Table 3 shows the *p* values of the Mann-Whitney U test on the relationships between clinical characteristics and DNA adducts in each tissue. Herb usage was associated with 5mdC and 5hmdC levels in the renal cortex. Although the number of herb users was very small in our cohort, as shown in the dot plots in Fig. 3, 5mdC and 5hmdC levels appeared to be reduced in the renal cortex of these patients. No significant differences were observed between smoking history and oxidative adducts, including 8-OHdA and 8-OHdG.

**Table 3:** Relationships between clinical characteristics and DNA adducts by tissue types

	5mdC	5hmdC	5fdC	dI	8-OHdA	8-OHdG
Renal cortex (*n* = 26)						
Sex (Male vs Female)	0.40	0.26	1.00	0.46	0.50	0.57
Smoking history (+) vs (−)*	0.77	0.39	1.00	0.97	0.50	0.86
Taking herbs (Yes vs No)	**0.041**	**0.005**	NA	0.44	1.00	NA
RCC (present vs absent)	0.18	0.82	0.80	0.23	0.33	1.00
Renal medulla (*n* = 21)						
Sex (Male vs Female)	0.70	0.64	NA	0.57	NA	NA
Smoking history (+) vs (−)	0.10	0.43	NA	0.085	1.00	0.20
Taking herbs (Yes vs No)	NA	NA	NA	NA	NA	NA
RCC (present vs absent)	0.51	1.00	1.00	0.70	1.00	0.40
Renal pelvis (*n* = 25)						
Sex (Male vs Female)	0.60	0.98	1.00	0.44	NA	0.84
Smoking history (+) vs (−)	0.73	0.69	0.40	1.00	NA	0.11
Taking herbs (Yes vs No)**	0.087	0.18	0.88	0.80	NA	0.44
UTUC (present vs absent)	0.08	**0.010**	1.00	0.89	0.67	0.38

**Fig. 4:**
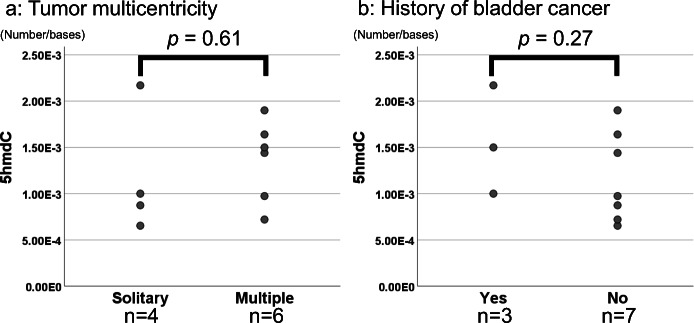
The comparison of 5hmdC in the pelvis-UTUC group. The molar ratios (number/bases) of 5hmdC were shown according to tumor multicentricity (**a**) and the presence of bladder cancer history (**b**). We described the sample size under the dot plots

5hmdC levels differed between the pelvis-UTUC group and pelvis-non-UTUC group (*p* = 0.010). The median and range of the molar ratio of 5hmdC in the pelvis-UTUC group were 1.2 × 10^− 3^ and 6.5 × 10^− 4^ to 2.2 × 10^− 3^, respectively, while those in the pelvis-non-UTUC group were 2.0 × 10^− 3^ and 6.7 × 10^− 4^ to 2.6 × 10^− 3^, respectively. Furthermore, we compared the quantity of 5hmdC according to the two clinical features, tumor multicentricity of UTUC and history of bladder cancer in the patients with UTUC and the *p* values were 0.61 and 0.27, respectively (Fig. 4).

**Fig. 5:**
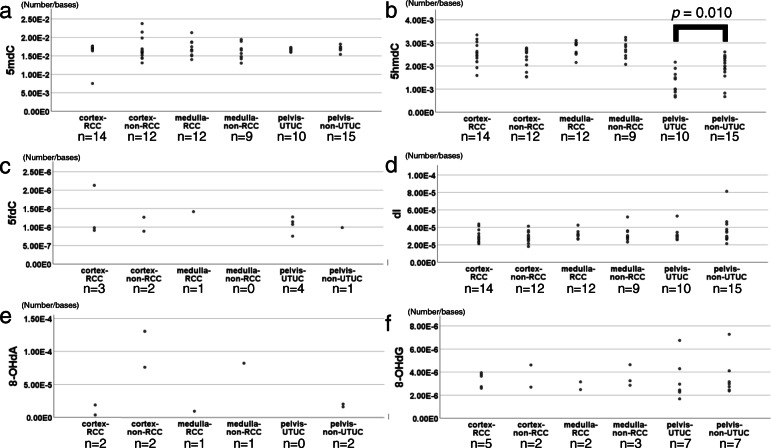
The molar ratio of DNA adducts in each group. The molar ratios (number/bases) of DNA adducts (**a**: 5mdC, **b**: 5hmdC, **c**: 5fdC, **d**: dI, **e**: 8-OHdA, **f**: 8-OHdG) on the y-axis were compared: Cortex-RCC group vs Cortex-non-RCC group; Medulla-RCC group vs Medulla-non-RCC group; Pelvis-UTUC group vs Pelvis-non-UTUC group. We described the sample size under the group name. As described in the main text and Table 3, 5hmdC levels were higher in the Pelvis-non-UTUC group than in the Pelvis-UTUC group (*p* = 0.010)

Figure 5 shows dot plots of the molar ratio of DNA adducts in the groups examined. The levels of the DNA adducts tested did not significantly differ between the cortex-RCC group and cortex-non-RCC group or between the medulla-RCC group and medulla-non-RCC group. RCC was not associated with the accumulation of specific DNA adducts in the renal cortex or medulla. Descriptive statistics, including the median, range, and standard deviation, of each DNA adduct according to the three tissue types are shown in Supplementary Table S7.

**Fig. 6:**
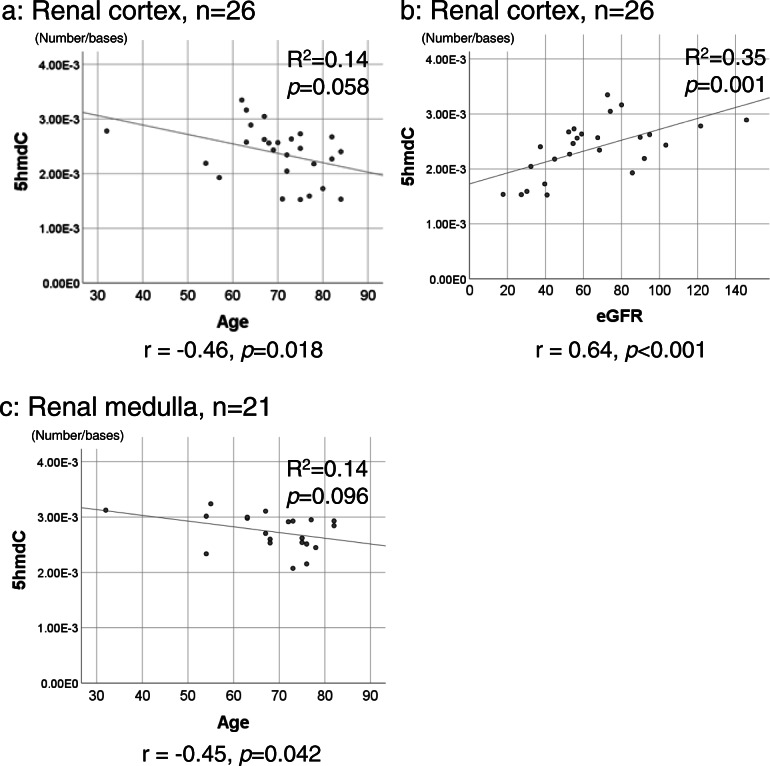
Relationships between 5hmdC levels, age, and eGFR. The molar ratios of 5hmdC in the renal cortex (**a** and **b**) and renal medulla (**c**) were plotted with age (**a** and **c**) and eGFR (**b**). The regression coefficient (*R*^2^) and *p* value were calculated by a linear regression analysis and indicated on the scatterplot with a regression line. The correlation coefficient and *p* value calculated by Spearman’s rank correlation analysis are described under the graphs

A relationship was observed between age and 5hmdC levels. Table 4 and Fig. 6 shows the relationships between DNA adducts, age, and eGFR. Specifically, age and eGFR correlated with 5hmdC levels in the renal cortex (r = − 0.46, *p* = 0.018 and *r* = 0.64, *p* < 0.001, respectively). Age also correlated with 5hmdC levels in the renal medulla (*r* = − 0.45, *p* = 0.042). However, neither 5mdC nor 5fdC correlated with age or eGFR in the renal cortex, renal medulla, or renal pelvis.

**Table 4:** Correlation analysis of age, eGFR, and DNA adducts by tissue types

	5mdC	5hmdC	5fdC	dI	8-OHdA	8-OHdG
Renal cortex (*n* = 26)						
Age	0.33	**0.018**	0.62	0.42	0.68	0.48
eGFR (mL/min)	0.61	**< 0.001**	1.00	0.79	0.80	0.65
Renal medulla (*n* = 21)						
Age	0.83	**0.042**	NA	0.64	NA	0.74
eGFR (mL/min)	0.70	0.091	NA	0.95	NA	0.75
Renal pelvis (*n* = 25)						
Age	0.32	0.94	0.75	.081	NA	0.83
eGFR (mL/min)	0.15	0.29	1.00	0.51	NA	0.65

**Fig. 7:**
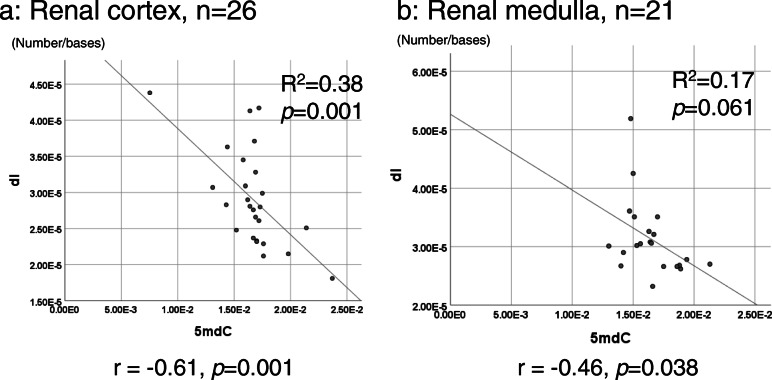
Relationship between 5mdC and dI. The relationships among all DNA adducts are shown in Supplementary Table S8. The molar ratios of 5mdC and dI in the renal cortex (**a**) and renal medulla (**b**) were plotted. The regression coefficient (*R*^2^) and *p* value were calculated by a linear regression analysis and indicated on the scatterplot with a regression line. The correlation coefficient and *p* value calculated by Spearman’s rank correlation analysis are described under the graphs

We also examined the relationships between all 6 DNA adducts (Supplementary Table S8) and found a negative correlation between 5mdC and dI in the renal cortex and medulla, but not in the renal pelvis (Fig. 7).

## Discussion

The present results revealed the presence of 5mdC, 5hmdC, 5fdC, dI, 8-OHdA, and 8-OHdG in the renal cortex, renal medulla, and urothelium of the renal pelvis. In our system, the adductomic approach theoretically facilitates the detection of multiple adducts other than those listed above. We previously identified several lipid peroxidation-induced DNA adducts, including butanone-etheno-2′-deoxyadenosine, in the gastric mucosa of gastric cancer patients [8]. We examined the chromatograms of all urological tissues for the retention times and m/z of these lipid peroxidation-derived DNA adducts; however, they were not found. Therefore, DNA adduct profiles appear to markedly differ between the stomach and urological organs [9].

dI is produced by cadmium and triptolide, which cause renal injury [29, 30]. Regarding carcinogenesis, Wang et al. reported the potential of ultraperformance LC/MS to detect lipid and purine metabolites in urine as differential markers of urothelial cancer from RCC, and among the potential biomarkers examined, they identified dI in urine [31]. However, in the present study, dI quantified in non-tumoral urinary tissue did not correlate with a malignant tumor status or eGFR.

8-OHdG and 8-OHdA are representative markers of DNA oxidative damage during oxidative stress [32]. Among urological cancers, a high level of 8-OHdG was detected in the RCC tissue and urine of patients with schistosomiasis-associated squamous cell carcinoma in the bladder [14, 16]. Recent studies proposed the use of 8-OHdG in urine to diagnose gastric carcinoma and lung cancer [33, 34]; however, only a few studies examined 8-OHdG levels in tissue as a potential biomarker of urological cancer in clinical settings [14, 15]. In the present study, 8-OHdG levels did not significantly differ between UTUC and non-UTUC patients. Oxidative agents, such as tobacco, directly damage the lungs and gastrointestinal system and are excreted in urine; therefore, 8-OHdG levels in urine may be useful as a biomarker of these oxidative agents. In the present study, 8-OHdG levels did not correlate with the urological cancer status, which implies that the urinary system is only indirectly exposed to tobacco carcinogens via urine. We also investigated the relationship between oxidative DNA adducts in urinary tissues and smoking habits because tobacco smoke has been shown to induce oxidative DNA damage in the lungs [35]; however, the results obtained revealed no significant differences in 8-OHdG or 8-OHdA levels between smokers and non-smokers. Using leukocyte DNA, Asami et al. and Besaratinia et al. investigated the relationship between 8-OHdG and smoking habits, but reported inconsistent findings [36, 37]. This discrepancy may have been due to leukocytes being indirectly exposed to tobacco smoke, in contrast to direct exposure in the lungs. The relationship between urinary 8-OHdG levels and smoking habits assessed by LC/MS is also controversial: Andreoli et al. reported a positive relationship [38], whereas Harman et al. found a negative relationship [39]. In the present study, oxidation-induced DNA adducts were not associated with smoking habits. The reasons for this result in our cohort may have been because we did not examine smoking habits in detail, such as quantity and duration, age is a confounding factor [37], and exposure to smoke in the urinary system is indirect. Further studies to compare DNA adducts in blood, urine, and urinary tissue and quantitatively evaluate smoking are needed in order to confirm the carcinogenicity of smoke-induced DNA adducts in the urinary system.

In the present study, 5hmdC levels in the non-tumoral renal pelvis were significantly lower in the UTUC group than in the non-UTUC group. However, no significant differences were observed in its precursor, 5mdC or its product, 5fdC, and another oxidation product, 5cadC was not detected. 5hmdC levels were previously reported to be reduced in many cancer tissues, including UC and premalignant lesions [25, 40–43]. 5mdC is oxidized and sequentially converted to 5hmdC, 5fdC, and 5cadC by ten-eleven translocation family proteins (TETs 1, 2, and 3). We previously reported that TET family protein levels were markedly lower in the tumoral portion than in the non-tumoral portion of the stomach [43], with TET1 playing an important role in the oxidation of 5mdC to 5hmdC. Foksinski et al. found a positive correlation between 5hmdC and TET1 transcript levels in malignant cell lines [44]. Furthermore, Dziaman et al. showed that 5hmdC and TET1 expression levels were significantly lower in colon cancer and colon adenoma, a premalignant lesion, than in normal colon tissue [41]. In addition, the silencing of TET1 reduced 5hmC levels and promoted cell proliferation by down-regulating the expression of AJAP1 in bladder cancer cells [45]. Another reason for the reduced levels of 5hmdC in malignant tissue may be a high cell proliferation rate. Jin et al. showed that 5hmC levels were reduced in Ki-67-positive cells using an immunohistochemical analysis [42]; the loss of 5hmC in tumors was attributed to an elevated rate of cell proliferation, which appeared to result in passive decreases in 5hmC levels in human tumor tissue. Previous studies also reported a negative linear correlation between the cell proliferation rate and global level of 5hmC in several mammalian tissues [46, 47]. As described above, 5hmdC levels are reduced by a high cell proliferation rate and low TET1 expression. We hereafter discuss the effects of reductions in 5hmdC levels in the non-tumoral tissue of UTUC patients, not RCC patients. Reductions in 5hmdC levels in the non-tumoral portion of patients with malignant tumors have been reported in other diseases. We previously demonstrated that the global levels of 5hmdC were lower in the non-tumor gastric mucosa of gastric cancer patients than in control gastric mucosa [10]. Other pathological changes in non-tumoral tissue have also been detected. Li et al. showed the clonal expansion of and somatic mutations in the normal urothelium of the non-tumoral tissue of UC patients [48]. Therefore, reductions in 5hmdC levels in UTUC patients may be a factor contributing to differences in the characteristics of cancer, particularly local recurrence. Since local recurrence after kidney-sparing surgery has been reported in 37–52% of UTUC patients [49], major guidelines recommend nephroureterectomy in many cases [50]. In contrast, local recurrence is rarer in patients with RCC, at a rate of 1–9% after partial nephrectomy and thermal ablation, while surgery robot-assisted partial nephrectomy recently achieved 0.5% [51, 52]. Therapeutic strategies for these two carcinomas markedly differ because of the risk of local recurrence. In the present study, we revealed reductions in 5hmdC levels in the non-tumoral tissue of UTUC patients, but not RCC patients. This result may reflect clinical differences in the two malignancies and, thus, reductions in the tissue levels of 5hmdC may be used for the risk stratification of the recurrence of UTUC. Additionally, we analyzed the relationships between the quantity of 5hmdC and the two clinical features, tumor multicentricity and the history of bladder cancer which are known as the prognostic factor of intravesical recurrence [53] in Fig. 4. However, the significant differences were not observed. A limitation of the present study is the small sample size. 5fdC, 8-OHdA, and 8-OHdG are rare DNA adducts in kidney tissue and the sample size were comparatively small in extended analysis about multicentricity and history of bladder cancer in patients with UTUC; therefore, investigations on a larger sample size are needed to reach concrete conclusions. Another limitation is that the control group included patients with other malignant tumors. For example, the cortex-non-RCC group included 8 UTUC patients, one ovarian cancer patient, and one sarcoma patient; therefore, the comparisons conducted in the present study were not necessarily between malignant tumor patients and non-tumor patients.

In the present study, herb usage appeared to contribute to reductions in 5hmdC levels in the renal cortex (Table 3). We did not extensively investigate all potentially detectable DNA adducts and their possible origins, particularly AA, in terms of urothelial carcinogenesis and kidney toxicity. The FDA issued a drug alert of the risk of AA in some of the herbs prescribed [54] and the herbs containing AA were not officially approved for prescription by Japanese government when outbreaks of AA related nephropathy occurred due to misidentification or private importation a decade ago in Japan [55]. In the present cohort, a large proportion of patients used Chinese herbal medicine; however, to the best of our knowledge and based on a detailed examination of clinical records, herbs such as kakkonto, daikenchuto, rikkunshito, daiokanzoto, and mashiningan, which are commonly prescribed in Japanese clinical practice, do not contain AA. We cannot conclude whether AA contributes to urothelial diseases in Japanese patients due to small number of patients investigated herein. Therefore, further studies on a larger number of patients are needed to clarify the effects of herb use on DNA methylation and hydroxymethylation.

In the present study, the status of RCC did not correlate with 5hmdC levels in the renal parenchyma. However, negative correlations were observed between age and 5hmdC levels in the renal cortex and medulla, as shown in Table 4 and Fig. 6. Therefore, the relationship between 5hmdC levels and aging appears to differ among organs. Previous studies reported age-dependent decreases in the global levels of 5hmdC in human T cells and the cerebral cortex [56, 57], whereas increases were detected in the cerebellum and sperm [58, 59]. Based on these findings, the mechanisms underlying 5hmdC reductions with aging warrant further study.

As shown in Fig. 6, eGFR positively correlated with 5hmdC levels, namely, 5hmdC levels decreased with the deterioration of renal function. Using a mouse system, Huang et al. reported down-regulation of the mRNA expression of TET1 and TET2 after ischemia-reperfusion injury, and subsequent reduction of global levels of 5hmdC in the kidney [60]. Furthermore, chronic hypoxia was shown to impair the activity of TET and alter the demethylation status in the mouse kidney [61]. Therefore, the low expression of TET may also lead to 5hmdC reductions with decreases in eGFR in humans.

Investigations to obtain a more comprehensive understanding of DNA adducts and modifications in urological systems are still in the burgeoning stage. The more extensive identification of other DNA adducts in more cases is needed. And the present study used LC/MS to detect the DNA adducts which is not widely available in a clinical practice. To apply the findings of DNA adducts acquired by LC/MS to a clinical setting, the investigation which evaluates the DNA adducts by immunohistochemistry staining is required.

## Conclusions

5hmdC levels in the normal renal pelvis were significantly lower in UTUC patients than in non-UTUC patients. Pathological changes also occurred in the normal urothelial mucosa, which may explain the higher risk of local recurrence in UTUC patients than in RCC patients.

## Supplementary Information

**Additional file 1 Supplementary Table S1**: Clinicopathological characteristics of cases. **Supplementary Table S2**: Standard compounds of DNA adducts and mass transition. **Supplementary Table S3**: Raw area data on DNA adducts in kidney samples. **Supplementary Table S4**: “Correction coefficient” and “normalized area” data on DNA adducts. **Supplementary Table S5**: The molar ratio of DNA adducts. **Supplementary Table S6**: Differences in the prevalence of DNA adducts by tissue types and the tumor status. **Supplementary Table S7**: Descriptive statistics of DNA adducts according to tissue types. **Supplementary Table S8**: A correlation analysis of DNA adducts.

## Data Availability

All processed files are presented in additional files. Raw data files are available upon request.

## Abbreviations

RCC: Renal cell carcinoma
UC: Urothelial carcinoma
UTUC: Upper urinary tract urothelial carcinoma
AA: Aristolochic acid
5mdC: C5-methyl-2′-deoxycytidine
5hmdC: C5-hydroxymethyl-2′-deoxycytidine
5fdC: C5-formyl-2′-deoxycytidine
dI: 2′-deoxyinosine
8-OHdA: C8-oxo-2′-deoxyadenosine
8-OHdG: C8-oxo-2′-deoxyguanosine
eGFR: estimated glomerular filtration rate
13C2-dC: 2′-deoxycytidine containing two carbon-13 atoms
N3-methyl-dC: N3-methyl-2′-deoxycytidine
13C2-dT: 2′-deoxythymidine containing two carbon-13 atoms
Etheno-dC: 3,N4-etheno-2′-deoxycytidine
13C2-dA: 2′-deoxyadenosine containing two carbon-13 atoms
5fdU: C5-formyl-2′-deoxyuridine
O2-methyl-dT: O2-methyl-2′-deoxythymidine
N3-methyl-dT: N3-methyl-2′-deoxythymidine
O4-methyl-dT: O4-methyl-2′-deoxythymidine
C5-Cl-dU: C5-chloro-2′-deoxyuridine
N6-methyl-dA: N6-methyl-2′-deoxyadenosine
N1-methyl-dA: N1-methyl-2′-deoxyadenosine
C2-hydroxy-dA: C2-hydroxy-2′-deoxyadenosine
N4-acetyl-dC: N4-acetyl-2′-deoxycytidine
13C2-dG: 2′-deoxyguanosine containing two carbon-13 atoms
O2-ethyl-dT: O2-ethyl-2′-deoxythymidine
N3-ethyl-dT: N3-ethyl-2′-deoxythymidine
O4-ethyl-dT: O4-ethyl-2′-deoxythymidine
C5-carboxy-dC: C5-carboxy-2′-deoxycytidine
Etheno-dA: 1,N6-etheno-2′-deoxyadenosine
N6-ethyl-dA: N6-ethyl-2′-deoxyadenosine
O6-methyl-dG: O6-methyl-2′-deoxyguanosine
N1-methyl-dG: N1-methyl-2′-deoxyguanosine
N2-methyl-dG: N2-methyl-2′-deoxyguanosine
C6-thio-dG: C6-thio-2′-deoxyguanosine
O4-nPr-dT: O4-normalpropyl-2′-deoxythymidine
O4-iPr-dT: O4-isopropyl-2′-deoxythymidine
C2-Cl-dA: C2-chloro-2′-deoxyadenosine
N4-CM-dC: N4-carboxymethyl-2′-deoxycytidine
O6-ethyl-dG: O6-ethyl-2′-deoxyguanosine
N1-ethyl-dG: N1-ethyl-2′-deoxyguanosine
N7-ethyl-dG: N7-ethyl-2′-deoxyguanosine
N2-ethyl-dG: N2-ethyl-2′-deoxyguanosine
N7-POB-dG: N7-pyridyloxobutyl-2′-deoxyguanosine
O4-nBu-dT: O4-normalbutyl-2′-deoxythymidine
N3-CM-dT: N3-carboxymethyl-2′-deoxythymidine
d4-N7-POB-dG: N7-pyridyloxobutyl-2′-deoxyguanosine containing four deuterium atoms
M1dG: 1,N2-malondialdehyde-2′-deoxyguanosine
N6-CM-dA: N6-carboxymethyl-2′-deoxyadenosine
4-OHE-dC: 4-oxo-2-hexenal-2′-deoxycytidine
DiEthyldG: Diethyl-2′-deoxyguanosine
O6-CM-dG: O6-carboxymethyl-2′-deoxyguanosine
N4-benzoyl-dC: N4-benzoyl-2′-deoxycytidine
N2-iBut-dG: N2-isobutyryl-2′-deoxyguanosine
Butanone-etheno-dA: Butanone-etheno-2′-deoxyadenosine
O6-benzyl-dG: O6-benzyl-2′-deoxyguanosine
4-ONE-dC: 4-oxo-2-nonenal-2′-deoxycytidine
Heptanone-etheno-dA: Heptanone-etheno-2′-deoxyadenosine
O2-POB-dT: O2-pyridyloxobutyl-2′-deoxythymidine
d4-O2-POB-dT: O2-pyridyloxobutyl-2′-deoxythymidine containing four deuterium atoms
NNN12: 2-[(3-Pyridyl)pyrrolidin-1-yl]-2′-deoxyinosine
4-ONE-dG: 4-oxo-2-nonenal-2′-deoxyguanosine
O6-POB-dG: O6-pyridyloxobutyl-2′-deoxyguanosine
